# Interplay of α-synuclein with Lipid Membranes: Cooperative Adsorption, Membrane Remodeling and Coaggregation

**DOI:** 10.1021/jacsau.3c00579

**Published:** 2024-03-19

**Authors:** Katarzyna Makasewicz, Sara Linse, Emma Sparr

**Affiliations:** † Department of Chemistry, Lund University, P.O. Box 124, SE-22100 Lund, Sweden; ‡ Division of Physical Chemistry, Center for Chemistry and Chemical Engineering, Lund University, P.O. Box 124, SE-22100 Lund, Sweden; ⊥ Biochemistry and Structural Biology, Lund University, SE-22100 Lund, Sweden

**Keywords:** α-synuclein, lipids, cooperativity, cooperative adsorption, lipid membrane remodelling, amyloid formation, protein−lipid coaggregation

## Abstract

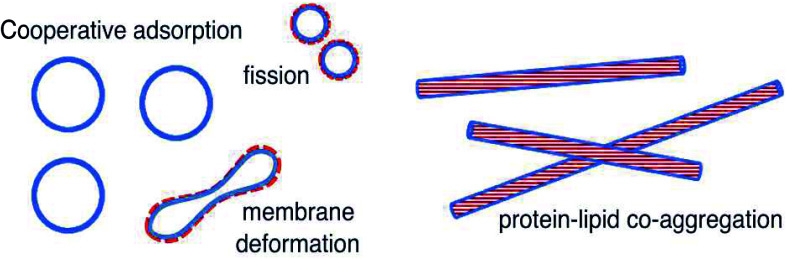

α-Synuclein is a small neuronal protein enriched at presynaptic termini. It is hypothesized to play a role in neurotransmitter release and synaptic vesicle cycling, while the formation of α-synuclein amyloid fibrils is associated with several neurodegenerative diseases, most notably Parkinson’s Disease. The molecular mechanisms of both the physiological and pathological functions of α-synuclein remain to be fully understood, but in both cases, interactions with membranes play an important role. In this Perspective, we discuss several aspects of α-synuclein interactions with lipid membranes including cooperative adsorption, membrane remodeling and α-synuclein amyloid fibril formation in the presence of lipid membranes. We highlight the coupling between the different phenomena and their interplay in the context of physiological and pathological functions of α-synuclein.

## Introduction

The lipid membranes of cells and organelles are abundant in proteins. The membrane-associated proteins can be categorized as integral or peripheral. The distinction between the two classes is based on their locations and the strength of their interaction with the membrane. Integral membrane proteins are mostly water-insoluble and interact strongly with the membrane core via hydrophobic interactions.^1^ They may serve as channels for transport of ions and small molecules and play a role in signal transduction.^2^ Peripheral membrane proteins generally have an overall high solubility in water and interact with one membrane leaflet through a variety of mechanisms including electrostatic interactions with the lipid headgroups and/or hydrophobic interactions with the acyl chains. Some peripheral proteins contain fatty acid chains, which anchor the protein to the membrane.^3^ The function of peripheral membrane proteins is believed to critically depend on the equilibrium between the free protein in solution and the membrane-bound protein. These proteins often play important roles in signaling, mediating interactions between membranes and cytoskeleton, blood coagulation, plasma membrane repair, lipid metabolism, and membrane remodeling events, including fusion and fission.^4^

This Perspective concerns α-synuclein, which is recognized as a peripheral membrane protein^5−9^ and for which an intricate interplay with membranes has been discovered over the recent years. We first introduce the context of physiological and pathological roles of α-synuclein *in vivo* and describe its structural features and the driving forces for its adsorption to lipid membranes. We then discuss two different aspects of monomeric α-synuclein membrane interactions: the cooperativity of the association and the α-synuclein-induced membrane deformation as well as the coupling between these events. Next, we discuss α-synuclein amyloid fibril formation in the presence of lipid membranes, relating the process of protein aggregation to the equilibrium between free and membrane-associated monomeric protein. The phenomena that we discuss are likely to be vital for understanding the physiological and pathological functions of α-synuclein, in both of which interactions with lipid membranes play a crucial role.

## α-Synuclein: Physiological and Pathological Functions

α-synuclein is expressed throughout the brain, but it is particularly enriched in presynaptic termini of neurons.^10^*In vivo*, it was shown to associate with synaptic vesicles,^11^ mitochondrial membranes,^12^ and plasma membrane^13^ and to interact with synaptic proteins including synapsin-III, synaptobrevin-2, VAMP2, and VMAT2.^14,15^ Based on its localization and association with synaptic vesicles, α-synuclein is hypothesized to be involved in neurotransmitter release. However, it remains controversial whether it inhibits or facilitates the process or whether it has a regulatory role of balancing the two effects. Importantly, α-synuclein is present only in vertebrates, which suggests that it is not strictly necessary for the functioning of the synapse. As a peripheral membrane protein, the function of α-synuclein is expected to critically depend on the equilibrium between its free and membrane-associated states and on the alterations of this equilibrium due to changes in environmental conditions.

α-Synuclein attracts a considerable amount of attention in the context of its aberrant aggregation associated with neurodegenerative diseases, most notably Parkinson’s Disease (PD). PD pathology is characterized by a progressive loss of dopamingeric neurons in substantia nigra, with the remaining neurons accumulating α-synuclein in pathological inclusions called Lewy Bodies.^16^ Initially, α-synuclein amyloid fibrils were thought to be the main component of Lewy Bodies. Later, it was recognized that these inclusions contain a large proportion of lipids,^17^ which motivates studies of α-synuclein amyloid formation in the presence of membranes. The importance of α-synuclein–membrane interactions in the context of both its physiological and pathological function is particularly interesting in the context of an unresolved question whether α-synuclein pathology is linked to the loss-of-function or gain-of-toxic function.^10^

## α-Synuclein Structure

**Figure 1:**
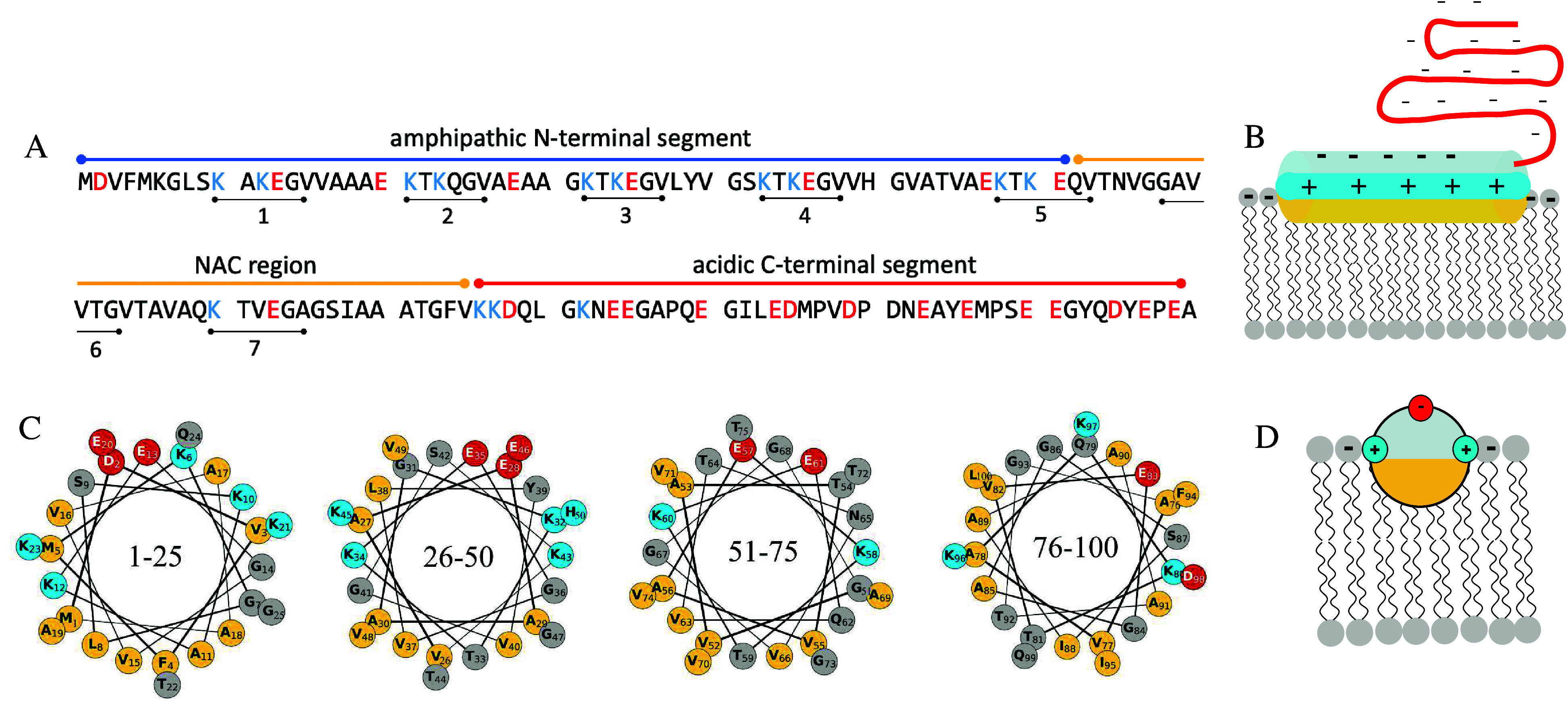
Structural features of α-synuclein. (A) Amino acid sequence. Acidic and basic residues are highlighted in red and blue, respectively. The seven repeats of the consensus sequence KTKEGV are marked below the sequence. (B) Cartoon showing the membrane-bound state of α-synuclein. The nonpolar face of the α-helix interacts with the acyl chain region of the lipid bilayer, positively charged residues interact with negatively charged lipid headgroups, while the highly negatively charged C-terminal segment remains in a disordered conformation. The locations of nonpolar, polar, acidic, and basic residues are indicated in yellow, gray, red, and blue, respectively. (C) Helical wheel projections of the 1–100 segment of α-synuclein sequence. The color coding is the same as in Figure 1B. The separation of the polar and nonpolar residues to the opposite faces of the helix becomes imperfect with increasing residue number. (D) Schematic cross section through the α-synuclein α-helix adsorbed to the membrane, using the same color coding as in panels B and C.

The α-synuclein sequence consists of 140 amino acids, and its characteristic feature is a highly asymmetric charge distribution (Figure 1A). The N-terminal segment of the protein (residues 1–60) is rich in positively charged residues. A region spanning residues 61–95 is rich in hydrophobic residues, and it forms the core of stacked β-sheets in α-synuclein amyloid fibrils. The segment 1–95 contains a recurring motif with the consensus sequence KTKEGV at an 11-residue periodicity, and mediates lipid membrane binding as shown *in vitro*(18,19) and *in vivo*.^20,21^ The C-terminal part of α-synuclein (residues 96–140) is highly negatively charged at neutral pH with a total of 15 acidic residues. The predominance of acidic residues leads to an upshift of the p*K*_a_ values and charge regulation upon fibril formation.^22^ Moreover, the intra- and intermolecular interactions, in particular between the oppositely charged termini, seem to be important in α-synuclein self-assembly, as studied using NMR spectroscopy, native mass spectrometry, and MD simulations^23−27^ and recently reviewed.^28^

The 1–95 segment of α-synuclein folds into an amphipathic α-helix upon adsorption to the lipid membrane, which can be observed as a change in the circular dichroism spectrum of the protein from one typical for random coil to a spectrum typical for α-helix.^18^ Studies using electron paramagnetic resonance (EPR) spectroscopy with spin labels^29^ or neutron reflectometry with contrast variation^5,30^ have provided information on the orientation and penetration depth of the protein in the membrane. These studies show that the helix is oriented parallel to the membrane interface and penetrates shallowly into the hydrophobic part of the lipid bilayer with its center located in the upper acyl layer just below the phosphate groups (Figure 1B).^5,29,30^

The amphipathic α-helix is an important structural motif of peripheral membrane proteins, such as exchangeable apolipoproteins and N-BAR proteins.^31−33^ However, the α-synuclein amphipathic α-helix has several distinct features. First, spanning approximately 100 residues, it is uniquely long. In other peripheral membrane proteins, the length of the amphipathic α-helix usually does not exceed 20–30 residues.^34^ In addition, the α-synuclein α-helix is characterized by a moderate hydrophobicity. The hydrophobic face consists mainly of alanines and valines, while in other peripheral membrane proteins the hydrophobic face typically consists of bulkier hydrophobic amino acids. Moreover, the hydrophobic face is also abundant in glycines, whose presence increases the entropic cost of forming the α-helix^35^ and is thus expected to destabilize the membrane-bound state of α-synuclein. The separation of the polar and nonpolar amino acids to the opposite faces of the helix becomes imperfect when going from the N-terminus to the C-terminus (Figure 1C).

The C-terminal segment of α-synuclein remains in a disordered conformation in the membrane-bound state. It extends into the solution, similar to a charged polymer brush (Figure 1B). The thickness of the brush layer was estimated to be approximately 6 nm in 110 mM NaCl in buffer solution at pH 7.4^5^ and was shown to decrease with increasing salt concentration,^30^ consistent with screening the electrostatic repulsion between the negatively charged side chains.

## Driving Forces for α-Synuclein Adsorption to Lipid Membranes

α-Synuclein adsorption to lipid membranes is governed by electrostatic and hydrophobic interactions. The electrostatic attraction between the negatively charged lipid headgroups and the positively charged N-terminal segment of the protein brings α-synuclein into the vicinity of the membrane. Numerous spectroscopy and microscopy studies have reveled that α-synuclein associates primarily with lipid membranes that contain anionic lipid species,^18^ although some studies have shown weak association also with zwitterionic membranes.^36,37^ In contrast to many peripheral membranes proteins, which for membrane association require specific lipid species (e.g., phosphatidylinositol 4,5-bisphosphate PIP(4,5)_2_),^38^ α-synuclein displays no clear lipid specificity, which was demonstrated experimentally for multiple lipid model membranes.^30,39^

α-Synuclein adsorption increases with increasing fraction of charged lipids up to a certain fraction, whereas for higher content of anionic lipids it reaches saturation and appears insensitive to variations in membrane charge density.^40^ The electrostatic attraction between negatively charged lipid headgroups and positively charged amino acids flanking the hydrophobic and hydrophilic faces of the α-helix likely stabilizes the membrane-bound state (Figure 1D). In addition, the electrostatic repulsion between the positively charged helices of adjacent adsorbed protein molecules may be screened at a negatively charged membrane. It has been shown for other peripheral membrane proteins interacting with lipid membranes through electrostatic interactions, e.g., cytochrome *c*,^41^ that negatively charged lipids are accumulated in the vicinity of the protein, which leads to depletion of charged lipid species from other areas of the membrane. This may also occur in the membrane with adsorbed α-synuclein.

The hydrophobic interactions between the exposed interior of the bilayer and the nonpolar face of the amphipathic helix are likely crucial for stabilizing the membrane-bound state of α-synuclein. Consistent with that, α-synuclein interaction with charged lipid membranes cannot be screened even at high salt concentrations.^18^ The importance of hydrophobic interactions in α-synuclein adsorption to membranes is emphasized by the fact that the protein shows higher adsorption to membranes with fluid hydrocarbon chains compared to gel state membranes with solid hydrocarbon chains.^42^ In the gel state membrane, the lipids are more closely packed, and the exposure of the hydrocarbon interior of the bilayer to the surrounding aqueous solution is reduced compared to the fluid membrane. Moreover, the α-synuclein adsorption was shown to decrease when going from DOPC:DOPS membranes to POPC:POPS membranes, while keeping membrane charge density and solution conditions the same.^30,36^ The decreased adsorption was explained by the decrease in the effective headgroup area when changing the hydrocarbon chain composition, which in turn implies lower exposure of the hydrophobic interior of the membrane.

The membrane-bound state of α-synuclein is destabilized by the electrostatic repulsion between the negatively charged C-terminal segments of the protein molecules bound to the membrane in the vicinity of each other as well as the repulsion between the C-terminal segments and the charged membrane surface. Altogether, the stability of the membrane-bound state and thus the equilibrium between the free and membrane-bound α-synuclein are governed by hydrophobic interactions and modulated by both attractive and repulsive electrostatic interactions.

## Cooperativity of α-Synuclein Adsorption to Lipid Membranes and Membrane Deformation

The interactions between α-synuclein and the lipid membranes are likely to play a crucial role in the physiological function of α-synuclein at the synapse. The proposed function of the protein in neurotransmitter release involves large-scale membrane remodeling events. Synaptic vesicles first fuse with the neuronal membrane to release their contents, then undergo fission and are later transported away for recycling. Below, we discuss two phenomena, cooperative binding and membrane deformation. The coupling between these two phenomena may be the key to the physiological function of α-synuclein.

## α-Synuclein Distribution in Excess of Membranes

**Figure 2:**
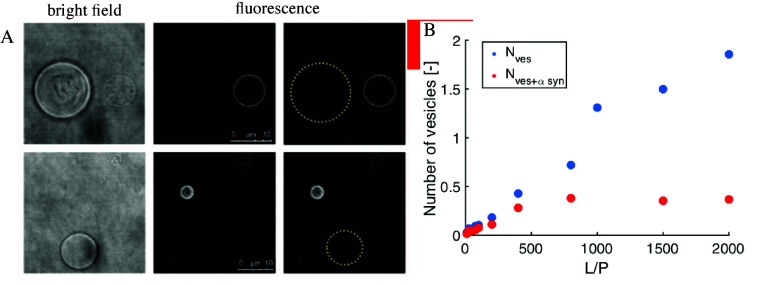
Characterization of α-synuclein association with lipid membranes in terms of binding cooperativity based on experimental observation of protein distribution in excess of lipid membranes. (A) Nonrandom distribution of α-synuclein in a population of giant unilamellar vesicles (GUVs). Two sets of bright-field (left) and fluorescence (middle) images of nonfluorescently labeled GUVs and fluorescently labeled α-synuclein. The protein association with GUVs renders them visible in the fluorescence images. The protein-free GUVs invisible in the fluorescence images are marked with a yellow dotted circle in the rightmost panel. (B) Distribution of α-synuclein in a population of small unilamellar vesicles studied with fluorescence correlation spectroscopy (FCS). Blue dots represent the total number of vesicles in the sample, while red dots represent the number of vesicles having α-synuclein bound. The numbers were extracted from the amplitudes of the FCS autocorrelation functions. Reproduced from ref (43). Copyright 2021 American Chemical Society.

Experiments using confocal fluorescence microscopy show that in the presence of excess giant unilamellar vesicles (GUVs), with diameter in the range of tens of μm, α-synuclein associates only with a subset of vesicles (Figure 2A).^43^ These observations could not be explained by curvature effects or inhomogeneities in the lipid composition. α-synuclein was also found to exhibit nonrandom distribution in a population of small unilamellar vesicles (SUVs), with diameter of approximately 100 nm, using fluorescence correlation spectroscopy (FCS) (Figure 2B). There are numerous literature reports consistent with these findings of nonuniform distribution of α-synuclein among lipid vesicles. Lee et al.^11^ analyzed the association of proteins with synaptic vesicles extracted from rat brain homogenate. α-synuclein was found to localize only to a subset of these vesicles, while synaptophysin and synaptobrevin were detected for all vesicles. Bureé et al.^15^ showed that α-synuclein molecules self-assemble on the surface of lipid vesicles into higher order structures, in which proteins could be cross-linked into groups of 8 and more while no cross-linking was observed in the absence of vesicles. Bureé et al. also carried out a FRET study with different combinations of acceptor and donor-labeled α-synuclein, which suggested that the protein molecules are organized on the SUVs as closely packed broken helices in an antiparallel arrangement.^15^ Similarly, Drescher et al.^44^ employed EPR spectroscopy and found that α-synuclein forms a supramolecular structure on the surface of a lipid membrane. The experiments revealed distinct distances between α-synuclein molecules bound to vesicles, as opposed to a homogeneous distribution of distances for a monomeric protein in solution. Importantly, the distance distributions measured were not affected by changing the lipid-to-protein (L/P) ratio from 250 to 1000, which would be the case if the supramolecular structures formed by proteins at lower L/P ratio would be diluted by adding more vesicles. A plausible explanation for the observations mentioned above is that α-synuclein adsorption to lipid membranes is characterized by strong positive cooperativity.

## Cooperative Phenomena

Cooperativity occurs when a binding event influences the equilibrium affinity for the next binding event of the same type of ligand to the same entity, be it a single macromolecule, a protein complex, or an extended surface. Cooperative binding enables the system to accomplish a regulatory effect over a narrower range of concentrations than in the case of independent binding, which is highly economical for a biological system. Examples of proteins that display cooperative ligand binding include hemoglobin, aspartate transcarbamoylase, threonine deaminase, and nicotinic acetylcholine receptor. These are proteins composed of multiple polypeptide chains; however, proteins composed of a single polypeptide chain may also display cooperative ligand binding, as for calcium binding to calmodulin^45^ and to proteins in the blood clotting system.^46^ These systems display a more or less well-defined stoichiometry of ligand binding. However, cooperativity is not limited to such cases and does not require the existence of a defined number of discrete binding sites. Surfactant micelle formation is an example of a cooperative self-assembly process. When the surfactant concentration in solution reaches a critical micelle concentration (CMC), micelles with a narrow size distribution start to form. When a small surfactant aggregate is formed and it grows by monomer addition, it becomes more and more favorable to add more monomers, because shielding of the hydrocarbon interior from the aqueous solvent becomes more effective with an increasing number of surfactant molecules in the micelle. However, the repulsion between the charged headgroups of the surfactants on the surface of the micelle limits aggregate growth. These driving forces result in the formation of micelles with a narrow size distribution (the aggregation number). Altogether, cooperativity operates on different organizational levels, from single protein chains to supramolecular structures.

## Cooperativity Formalism

Small molecule binding to proteins is usually deduced as cooperative or independent based on the shape of the binding curve, with cooperative binding giving rise to a steeper dependence of fractional saturation on ligand concentration.^47^ The same holds true for systems with multiple binding events, such as the association of proteins with lipid membranes. A study of α-synuclein adsorption to a supported lipid bilayer using total internal reflection fluorescence microscopy (TIRF) revealed that the increase in density of membrane-bound protein as a function of total protein concentration is steeper than expected for independent binding (Figure 4).^43^

**Figure 3:**
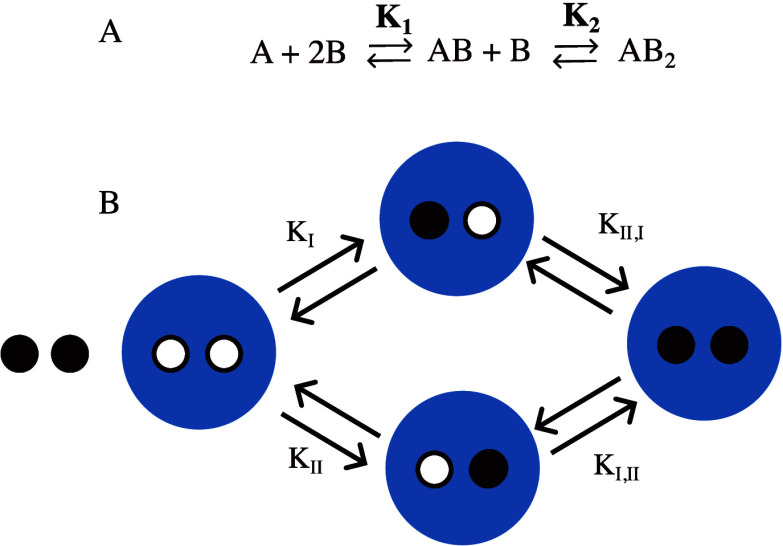
Cartoon showing binding equilibria described on a macroscopic level (A) and on a microscopic level (B) for a macromolecule (blue) with two binding sites for the same ligand (black). *K*_I_, *K*_II_, *K*_I,II_. and *K*_II,I_ are microscopic binding constants, while *K*_1_ and *K*_2_ are macroscopic binding constants.

A direct way to describe cooperativity is in terms of the free energy coupling between the binding events, ΔΔ*G*. The macroscopic description of the system involves specification of the concentrations at each occupancy level and involves macroscopic binding constants, *K*_1_ and *K*_2_, etc., which relate to the binding of the first and second ligand, etc. but do not differentiate between the different binding sites (Figure 3A). The macroscopic binding constants can be used to calculate ΔΔ*G*.

In a simple case of two coupled binding events, the equation takes the form:
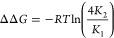
1where 4 is a statistical factor due to the fact that there are more configurations available for the first bound compared to second bound protein. In the general case of *N* coupled binding events, the free energy coupling between subsequent binding steps *n* – 1 and *n* is given as
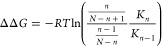
2When ΔΔ*G* < 0, cooperativity is defined as positive, which is manifested in ligand binding becoming more favorable when other ligands are bound. When ΔΔ*G* > 0, the cooperativity is defined as negative, and ligand binding makes binding of additional ligands less favorable. ΔΔ*G* = 0, corresponds to independent binding.

The description of the system on a macroscopic level is sufficient when all of the binding sites are identical (Figure 3A). A description on a microscopic level differentiates between binding events to the different binding sites (Figure 3B) and is required if there is a coexistence of binding sites with different intrinsic affinities.

In the context of α-synuclein adsorption to lipid membranes, the concept of a binding site may be misleading. As discussed above, since the protein does not require any specific lipid species for membrane association, we expect that as long as the content of negatively charged lipids in the fluid membrane is sufficient, all places on the membrane are equivalent for protein adsorption. On the other hand, α-synuclein adsorption is expected to affect the local curvature and/or may perturb the thermal fluctuations of the membrane.^48^ It may also induce a heterogeneous lipid distribution in a fluid membrane: since the interactions between positively charged side chains of α-synuclein and negatively charged lipid headgroups stabilize the membrane-bound state, the charged lipids are expected to cluster around the bound proteins. Thus, a place on a membrane with adsorbed α-synuclein is expected to have different properties than a place devoid of bound protein, and this may affect the adsorption of additional protein molecules.

**Figure 4:**
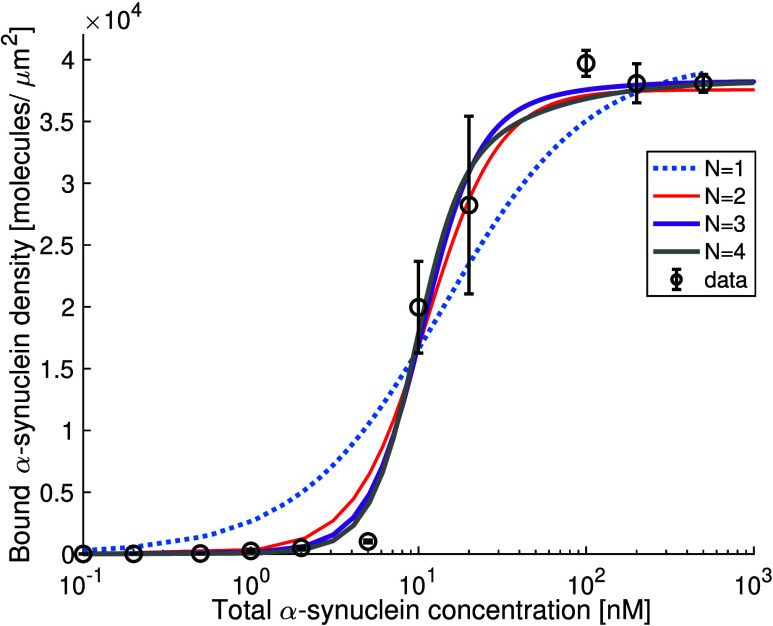
Density of α-synuclein bound to a supported lipid bilayer measured with a Total Internal Reflection Fluorescence (TIRF) Microscopy. The dotted blue line shows a fit to the Adair eqaution^47^ assuming one binding site, which corresponds to independent binding. The red, purple, and gray lines show fits to Adair equations assuming two, tree, and four coupled binding sites, respectively. N is the number of coupled binding sites. Reproduced from ref (43). Copyright 2021 American Chemical Society.

Because the intrinsic affinity of α-synuclein for the bare membrane is the same all over the membrane, a macroscopic level description is sufficient. This approach was used to model the binding curve of α-synuclein to a supported lipid bilayer measured with TIRF microscopy (Figure 4). Notably, a biologically relevant level of cooperativity per binding step, −8 kJ/mol, was enough to fit the experimental data.^43^ Such value is consistent with the ΔΔ*G* values known for other systems involving cooperative binding.^49^ Increasing the free energy coupling per binding event above approximately −10 kJ/mol results in very little narrowing of the total ligand concentration over which the regulatory effect can be accomplished, and thus, there is no evolutionary pressure to enhance the strength of the interactions leading to cooperative binding beyond this limit.^49^

## Molecular Origin of Cooperative α-Synuclein Binding to Membranes

The nonrandom distribution of α-synuclein at the membrane implies that it is more favorable for a protein molecule to bind next to another one compared to at a bare membrane surface. Such cooperative binding (ΔΔ*G* < 0) could have an entropic (ΔΔ*S* > 0) and/or enthalpic (ΔΔ*H* < 0) origin. The nonrandom distribution of the protein is clearly unfavorable in terms of an entropy cost for the protein; however, this may be counterbalanced by a lower entropy cost for the lipids if the protein is bound in clusters rather than spread out over the membrane. The clustering of the protein would thus be favored because the lipids retain a larger number of possible configurations compared to the independent binding case. Such effect would be analogous to depletion forces in solutions of coexisting large and small particles, where larger particles cluster to give the smaller ones, and thereby the system as a whole, a larger number of possible configurations.^50^ An enthalpic coupling between the binding events may emerge from protein–protein, lipid–lipid, protein–lipid, protein–solvent, and/or membrane–solvent interactions. For example, attractive interactions between the amphipathic helices packed closely on the membrane surface may play a role, as was shown to be the case for an N-BAR protein endophilin.^51^

Membrane-mediated forces may also play a role. It was shown that membrane-mediated long-range repulsive forces may lead to protein ordering on lipid membranes.^52^ Moreover, damping of the thermal fluctuations of the membrane upon protein adsorption may lead to an effective attractive interaction between proteins, as shown for shiga toxin clustering on lipid membranes.^53^ This so-called thermal Casimir effect can be considered a generic clustering mechanism operating between sufficiently rigid inclusions tightly interacting with the membrane. We note that it is unclear whether the membrane-bound amphipathic α-helix is sufficiently rigid for this effect to play a significant role.

## Coupling of Cooperativity and Membrane Deformation by α-Synuclein Adsorption

**Figure 5:**
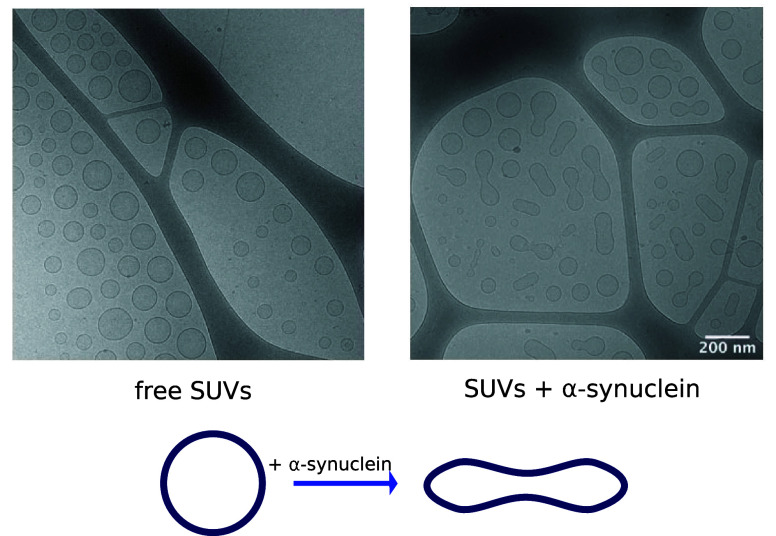
α-Synuclein adsorption to spherical small unilamellar vesicles (SUVs) induces their deformation to spheroids or pearl-on-a-necklace structures. Cryo-TEM images of SUVs composed of DOPC:DOPS 7:3 without (left) and with α-synuclein at the L/P ratio 200 (right). Reproduced with permission from ref (57). Copyright 2022 Cambridge University Press.

The adsorption of α-synuclein to lipid membranes has been shown to cause membrane remodelling.^54−57^ When α-synuclein is added to a sample of SUVs of approximately 100 nm in diameter, the SUVs deform to ellipsoids or pearl-on-a-string structures (Figure 5). The direction of deformation is consistent with α-synuclein adsorption, leading to an increase in the spontaneous curvature of the membrane.

There exist a multitude of ways through which proteins can generate spontaneous curvature in lipid membranes.^58^ Below, we briefly discuss the mechanisms that might be relevant for the case of α-synuclein, including nonspecific effects due to adsorption, the insertion of an amphipathic helix, and entropic (steric + electrostatic) repulsion of the unstructured domains of membrane-associated proteins.

The adsorption of macromolecules to a lipid membrane generally leads to a change in the spontaneous curvature, provided that there is an asymmetry in composition between the two leaflets of the bilayer.^59^ The higher the asymmetry, the greater the effect on the induced curvature, which can lead to membrane deformation. Although membrane deformation by adsorption is a general mechanism and does not depend on the specific structural features of the adsorbant, an amphipathic helix is a common structural motif of membrane-deforming proteins.^58,60^ An amphipathic helix induces membrane curvature by acting as a wedge. The shallow insertion of the helix into the bilayer results in an increase in the effective lateral area of the headgroups. The curvature induction relieves the resulting area mismatch of the monolayers.

The highly negatively charged C-terminal segment of α-synuclein remains unstructured in the membrane-bound state. The C-terminal segments of neighboring proteins on the membrane will repel each other electrostatically and sterically, which is expected to determine the distance between the membrane-bound α-synuclein molecules. Recently, steric repulsion between adsorbed molecules (referred to as crowding) was proposed as an additional mechanism of membrane deformation by proteins containing unstructured segments, where the membrane would curve to allow the disordered domains to be further apart and thus increase their conformational entropy.^61,62^

Efficient membrane remodeling requires proteins to accumulate in spots on the membrane, so that the effects of individual proteins add up. At this point, it is highly relevant to consider the coupling between membrane deformation and cooperative adsorption. Cooperative association with lipid membranes was suggested to play an important role in the membrane deformation by amphipathic helices, where insertion of the first helix favors insertion of the subsequent ones, leading to clustering of the proteins in specific regions of the membrane and exerting a strong effect on the local membrane topology.^60^

The coupling between cooperative binding and membrane deformation is also manifested in membrane-mediated protein–protein interactions. Braun et al, employed molecular dynamics simulations to show that the effect on local membrane curvature of an adsorbing amphipathic helix can be highly anisotropic for a long helix as in the case of α-synuclein.^48^ The anisotropy of the local curvature induced by a single membrane-associated α-synuclein molecule was suggested to lead to a specific alignment of the associated proteins and protein ordering on the membrane. This may result in a mutual reinforcement of the effects of protein adsorption and membrane curvature and therefore can provide an effective mechanism of curvature generation.

Regardless of its molecular origin, clustering of the protein on the membrane as opposed to a random distribution results in locally high protein concentration even at low total protein concentrations, which may have a substantial effect on the membrane. A membrane patch covered with α-synuclein molecules acquires spontaneous curvature that deviates from the bare membrane, which causes the membrane to bend. Thus, the cooperative adsorption of α-synuclein to lipid membranes may induce deformation in distinct spots on the membrane. Importantly, the magnitude of the spontaneous curvature generated by adsorbed α-synuclein may change as a result of changes in solution conditions, solute concentrations, or lipid composition, which may be relevant to the regulation of the membrane remodeling processes at the synapse.

Cooperative protein adsorption to lipid membranes can also be conceptualized as the formation of a supramolecular structure on the membrane surface, which goes well in hand with membrane deformation in various biological processes.^63−66^ Multiple peripheral membrane proteins were shown to form 2D arrays on the surface of lipid membranes as in the case of annexins^67^ and BAR-domain proteins.^51^ For these proteins, the biological functions are dependent on the emergent properties of the supramolecular structures they form and range from regulation of blood coagulation (annexin a5^67^) to membrane deformation at different stages of endocytosis (BAR-domain proteins^68^).

## Membrane Fluidity and Curvature Effects

The spontaneous bending of the membrane upon α-synuclein adsorption, as shown in Figure 5, implies that the protein-covered and bent membrane corresponds to a lower energy state than that of the protein-covered and unbent membrane. As a result, it is expected that α-synuclein adsorption to nondeformable membranes, such as membranes in gel phase or supported lipid bilayers, will be less favorable than the adsorption to deformable fluid membranes. This is consistent with experimental observations.^40,42^ It has been shown that α-synuclein associates with membranes in fluid state, while much less association has been observed for gel phase membranes with solid hydrocarbon chains.^42^ The fluid and the gel state membranes are clearly different with respect to both the hydrophobicity of the membrane interface (see Driving Forces for α-Synuclein Adsorption to Lipid Membranes) and the lipid dynamics, in particular the lateral diffusion of the lipids in the membrane. The rearrangement of the adsorbed protein to an optimal state and the possible supramolecular ordering is likely facilitated at the fluid interface, in analogy to previous findings for other biomacromolecules and particles that form dense clusters at lipid membranes.^69−71^ Altogether, the more favorable adsorption of α-synuclein to fluid membranes is likely due to a combination of larger hydrophobic core exposure in fluid membranes, the fact that they can deform, and the fact that the protein molecules are able to achieve the optimal arrangement on the membrane through lateral diffusion.

Since α-synuclein adsorption to a lipid membrane induces spontaneous curvature, it is expected that its adsorption will be more favorable to vesicles whose size matches the preferred curvature of the adsorbed protein. Such an energetic preference may be negligible for an individual protein molecule, but the effect may add up to be significant in cases where there is a large number of proteins covering the same vesicle. It is therefore possible that the high positive cooperativity may cause higher affinity of the protein to smaller vesicles compared to larger ones. Indeed, there are reports of α-synuclein having higher affinity for vesicles of diameter below 40 nm when compared with vesicles with diameter of 100 nm or more.^18,36,37,72^ In the case of larger vesicles, there is a driving force to match their curvature to the spontaneous curvature of the membrane with an adsorbed α-synuclein layer, which leads to membrane remodeling. Thus, deformation toward higher spontaneous curvature of larger vesicles and higher affinity for the smaller vesicles are in fact two manifestations of the same phenomenon.

The discussion above concerns α-synuclein-induced deformation of SUVs, with sizes in the nm range. α-Synuclein was also shown to remodel membranes of GUVs with sizes in the μm range^54^ and in these cases it was manifested as tubulation. This can be understood by comparing the length-scales involved: the deformation of a GUV to a prolate does not bring the membrane any closer to the preferred curvature of the α-synuclein-covered membrane, as a prolate of size in the μm range would also appear flat to the protein, whereas the curvature of the protruding tubules is in a similar range as the preferred curvature.

## On the Way to Fission

The effect of protein adsorption on the spontaneous curvature of the membrane may be modulated by the lipid composition of the membrane. The DOPC:DOPS SUV deformation as shown in Figure 5 persists for at least 24 h,^57^ which suggests that the deformed structures are kinetically stable. Such kinetic stability is expected to be due to a high energy barrier for fission related to the disruption of membrane continuity, which may not be overcome by thermal fluctuations for the studied system within the experimental time frame. The height of the energy barrier between the fission intermediate and the fission products can be modulated not only by the protein composition of the membrane but also by the lipid composition. The DOPC and DOPS lipids have symmetric cylindrical shapes and thus their equilibrium state is a lamellar phase.^73^ It is reasonable to assume that the inclusion of lipid species favoring highly curved geometries may facilitate vesicle fission due to α-synuclein adsorption. Indeed, Andersson et al. used a single-vesicle fluorescence microscopy assay to show that the addition of α-synuclein to vesicles that also contain ganglioside GM1 (a micelle-forming lipid) leads to vesicle fission, which was not observed for the corresponding ganglioside-free system with same content of anionic lipids.^74^ This finding may be relevant to the physiological function of the protein, since ganglioside lipids are abundant in neuronal membranes.^75^

## α-Synuclein Amyloid Formation in the Presence of Lipid Membranes

**Figure 6:**
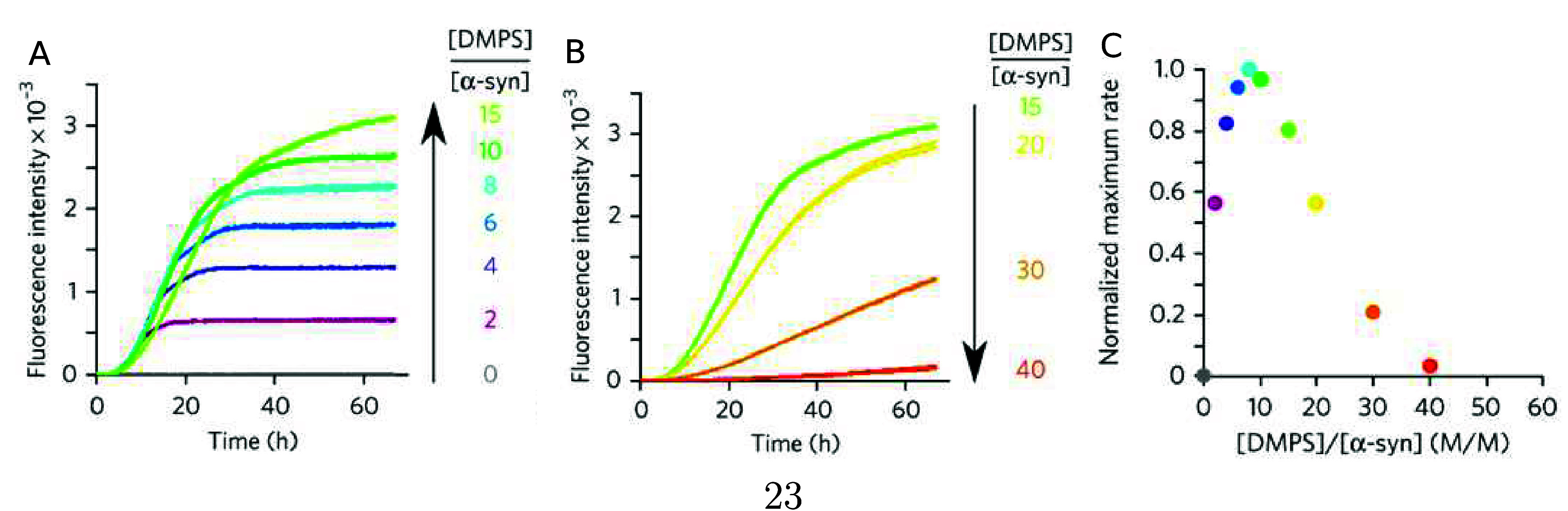
α-Synuclein amyloid formation in the presence of lipid membranes. (A,B) Lipid membranes composed of DMPS accelerate α-synuclein amyloid formation at a low L/P ratio and inhibit at a high L/P ratio. (C) Dependence of the maximum rate of α-synuclein aggregation with changes in the lipid to protein ratio. Adapted with permission from ref (77). Copyright 2015 Springer Nature America, Inc.

The effect of lipid membranes on α-synuclein aggregation ranges from acceleration to inhibition depending on the solution conditions, membrane composition and the relative concentrations of protein and lipids.^76−79^ In a system consisting of small unilamellar vesicles and initially monomeric α-synuclein, aggregation is accelerated in the conditions of protein excess over the membrane (low L/P ratio), where monomers in solution and membrane-bound protein coexist (Figure 6A,C). In contrast, aggregation is inhibited in the conditions of membrane excess (high L/P ratio), where the membrane-bound form dominates and the free protein concentration is very low (Figure 6B,C). The dependence of the effect of membranes on α-synuclein aggregation suggests that for the aggregation to occur, free and vesicle-associated protein needs to coexist. Galvagnion et al.^77^ showed that the rate of primary nucleation in α-synuclein amyloid fibril formation can be increased by 3 orders of magnitude in the presence of negatively charged lipid membranes in the low L/P ratio conditions. Such an enhancement of the primary nucleation rate has been ascribed to an increased local concentration of α-synuclein on the membrane and a shift in the conformational space of the protein to an ensemble of conformations compatible with the nucleation process. To better understand the mechanism of α-synuclein fibril formation in the presence of lipid membranes, it may be necessary to complement the structural biology and chemical kinetics with a thermodynamic perspective. Describing the free energy landscape of the system including all protein states (monomer, membrane-bound, amyloid fibril, etc.) and the heights of the energy barriers separating them may provide important insights into the mechanism of membrane-assisted aggregation of α-synuclein. Below, we discuss the results of studies of the equilibrium between membrane-bound and free α-synuclein as a function of the L/P ratio, which reveal crucial differences in the behavior of the system on a molecular level between conditions, which on a macroscopic level result in the acceleration or inhibition of α-synuclein amyloid formation.

## The Roles of Free and Membrane-Bound α-Synuclein in Amyloid Formation

The segment spanning residues 1–95 of α-synuclein folds into an amphipathic α-helix upon membrane binding. However, this is not an all-or-nothing transition: α-synuclein can engage in membrane interactions with shorter stretches of this segment.^80−82^ The first experimental evidence of the existence of multiple membrane-binding modes of α-synuclein was provided by Bodner et al.^80^ for a 5:3:2 DOPE:DOPS:DOPC membrane. The authors showed that at low L/P ratios, apart from α-synuclein molecules associated with the membrane with the full membrane-binding segment, a fraction of protein molecules interacts with the membrane with a short N-terminal segment (residues 1–25). The relative populations of protein interacting with the membrane with sequence segments of different length were shown to depend on the lipid to protein ratio. At high amount of lipid membrane area, only the fully bound protein is present in the sample. At low L/P ratio, both the long and short binding modes as well as free protein coexist.

The observations of different binding modes upon L/P variation may be related to the conditions for which the presence of vesicles induces protein aggregation. At low L/P ratio, α-synuclein amyloid aggregation is accelerated as compared to the membrane-free system.^26,77^ Since the amyloid fibrils in such cases may form through heterogeneous primary nucleation on the surface of the membrane, , or elsewhere, it may be reasonable to speculate that the specific properties of the membrane at these conditions are responsible for lowering the free energy barrier separating the monomer from the fibrillar state. At high L/P ratio, where the presence of membranes inhibits α-synuclein aggregation, the equilibrium is strongly shifted toward the fully membrane-bound form of the protein, and the free protein concentration is very low. In such a way, the fully membrane bound state, where the hydrophobic residues in segment 61–95 interact with the membrane and are shielded from the solvent and from each other, can be considered as having an aggregation-protecting role. Thus, in the L/P ratio regimes where lipid membranes have a distinctly different effect on α-synuclein amyloid formation, the protein behaves differently on the molecular level.

In summary, the difference between the protein excess and membrane excess conditions is the overall stronger α-synuclein interaction with the membrane in the latter. Thus, any factors affecting the α-synuclein-membrane interaction strength may trigger a change from nonaggregating to aggregating conditions. The strength of α-synuclein-membrane interaction can be modulated by temperature, pH, ionic strength,^18^ changes in the lipid composition and fluidity of the membrane,^36,37,42,72,83^ point mutations,^72,81,84^ and post-translational modifications (PTMs).^85−87^ Interestingly, all Parkinson’s Disease-associated point mutations (A30P, E46K, H50Q, G51D, A53E, A53T) are localized in the membrane-binding segment of α-synuclein and not in the NAC-domain, which forms the core of α-synuclein amyloid fibrils. This provides additional argument that aberrant aggregation of α-synuclein in Parkinson’s Disease may be linked to perturbations of the equilibrium between the free and membrane-bound state and not directly to higher aggregation propensity of the disease related mutants. The effect of PTMs, including N-terminal acetylation, phosphorylation, ubiquitination, SUMOylation, nitration and truncations on α-synuclein-membrane interaction are reviewed in ref (88).

**Figure 7:**
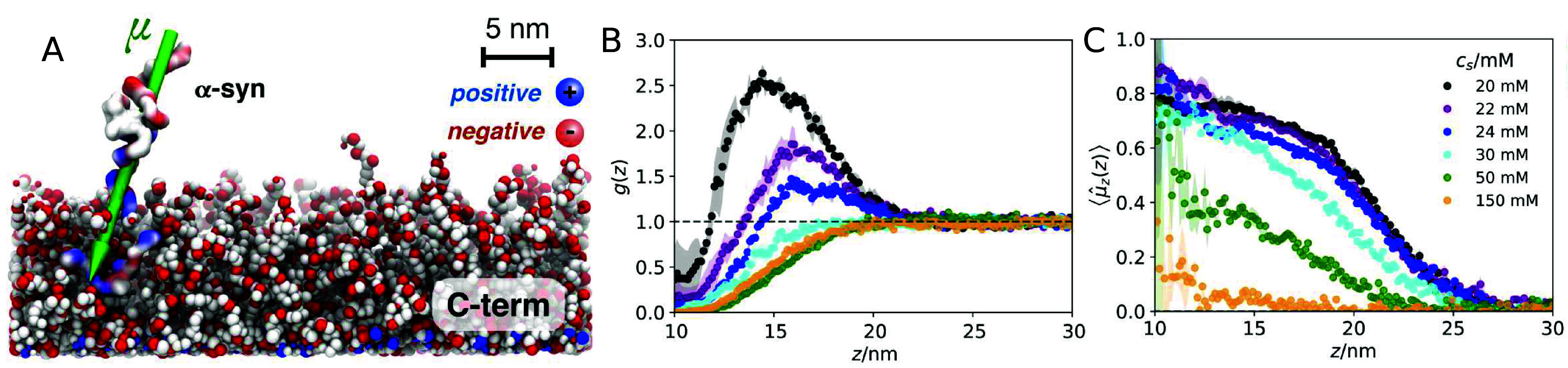
Interaction of an α-synuclein monomer and a surface covered with C-terminal segments of the protein. (A) Snapshot from Monte Carlo simulation at low ionic strength revealing a preferential orientation of α-synuclein monomer with respect to the protein-covered surface, where the positively charged N-terminal segment of the monomer interacts with the negatively charged C-terminal segments on the surface. (B) Monomer mass center distribution as a function of the distance from the surface, *g*(*z*), for ionic strength conditions from 0 to 140 mM NaCl. The peak in *g*(*z*) at low ionic strength indicates the presence of an attractive intermolecular interaction. At high ionic strength, *g*(*z*) < 1, indicates that the monomer is repelled from the surface.. (C) Average orientation of the α-synuclein monomer dipole as a function of the distance from the surface for different values of ionic strength. Decrease in ionic strength leads to stronger alignment of the monomer dipole with respect to the surface. Reproduced with permission from ref (26). Copyright 2020 Cambridge University Press.

An interesting question concerns the role of the protein-covered lipid membrane in the amyloid formation process. In this context, an important clue may come from the dependence of the effect of DOPC:DOPS membranes on α-synuclein aggregation as a function of ionic strength and the decreased rate of fibril formation upon an increase in the NaCl concentration from 0 to 140 mM.^26^ The fact that heterogeneous primary nucleation of a net-negatively charged protein on a net negatively charged surface slows with increasing salt concentration may seem counterintuitive. However, the highly asymmetric charge distribution in the α-synuclein sequence, with a high abundance of positively charged residues in the N-terminal segment and negatively charged residues in the C-terminal segment, is expected to play a role. The attractive interaction that leads to membrane-induced α-synuclein fibril formation may originate from the electrostatic attraction between the positively charged N-terminal segment of the free α-synuclein monomer and the negatively charged C-terminal segments of the membrane-associated proteins. To test this hypothesis Gaspar et al, performed Monte Carlo simulations, where the interaction between an α-synuclein monomer and a surface grafted with α-synuclein C-terminal segments was studied at different salt concentrations.^26^ A snapshot from the simulation at low ionic strength is shown in Figure 7A. The α-synuclein mass center distribution function with respect to the surface, g(z), extracted from the simulations (Figure 7B), shows that at high salt, the monomer is repelled from the surface. This can be explained with a decrease in the conformational entropy of both the monomer and the C-terminal segments as the monomer approaches the interface. However, at low ionic strength, the g(z) shows the presence of an attractive interaction between the monomer and the C-terminal segments that dominates over the electrostatic repulsion. The average orientation of the α-synuclein dipole as a function of the distance to the surface revealed a strong orientation of the monomer with respect to the surface at low salt (Figure 7C), with the positively charged N-terminal part of the free monomer embedded in the negatively charged layer of the C-terminal segments on the surface (as shown in Figure 7A). At higher ionic strength, no particular orientation of the α-synuclein monomer dipole is favored (Figure 7C, yellow). Thus, the attractive electrostatic interaction and the resulting alignment of an α-synuclein monomer with respect to the C-terminal segments of the membrane-bound protein molecules are expected to play important roles in amyloid formation in the presence of membranes. Importantly, these results suggest that it is the protein-covered membrane surface rather than the bare membrane that has a catalytic effect on α-synuclein amyloid fibril formation.

## The Role of Lipid Properties in α-Synuclein Amyloid Fibril Formation

Galvagnion et al. studied the effect of membranes composed of different lipid species on α-synuclein aggregation and showed that lipid properties such as the length of the hydrocarbon chains play a crucial role.^42,77^ The authors showed that α-synuclein associates with lipid membranes with fluid hydrocarbon chains of different compositions (DLPS, DMPS, DOPS). However, only membranes composed of the lipid species with the shortest acyl chains, DLPS and DMPS, induced α-synuclein aggregation at pH 6.5. Interestingly, Gaspar et al. showed that membranes composed of lipid species with longer acyl chains (DOPC:DOPS) induce α-synuclein aggregation at pH 5.5.^26^

The process of α-synuclein amyloid formation in the presence of DLPS membranes (12 carbon long acyl chains) is faster than in the presence of DMPS membranes (14 carbon long acyl chains).^42^ This can be compared to the differences in the aqueous solubility of these lipids, which scales with the length of the acyl chains and is 100 and 10 nM for DLPS and DMPS, respectively. The fact that DLPS membranes cause a greater acceleration of α-synuclein aggregation may indicate that at least part of the free energy barrier for the aggregation process in these model systems is related to the transfer of lipid molecules from lipid-rich environment (the membrane) to a protein-rich environment (the aggregate), a process that most likely depends on lipid solubility.

**Figure 8:**
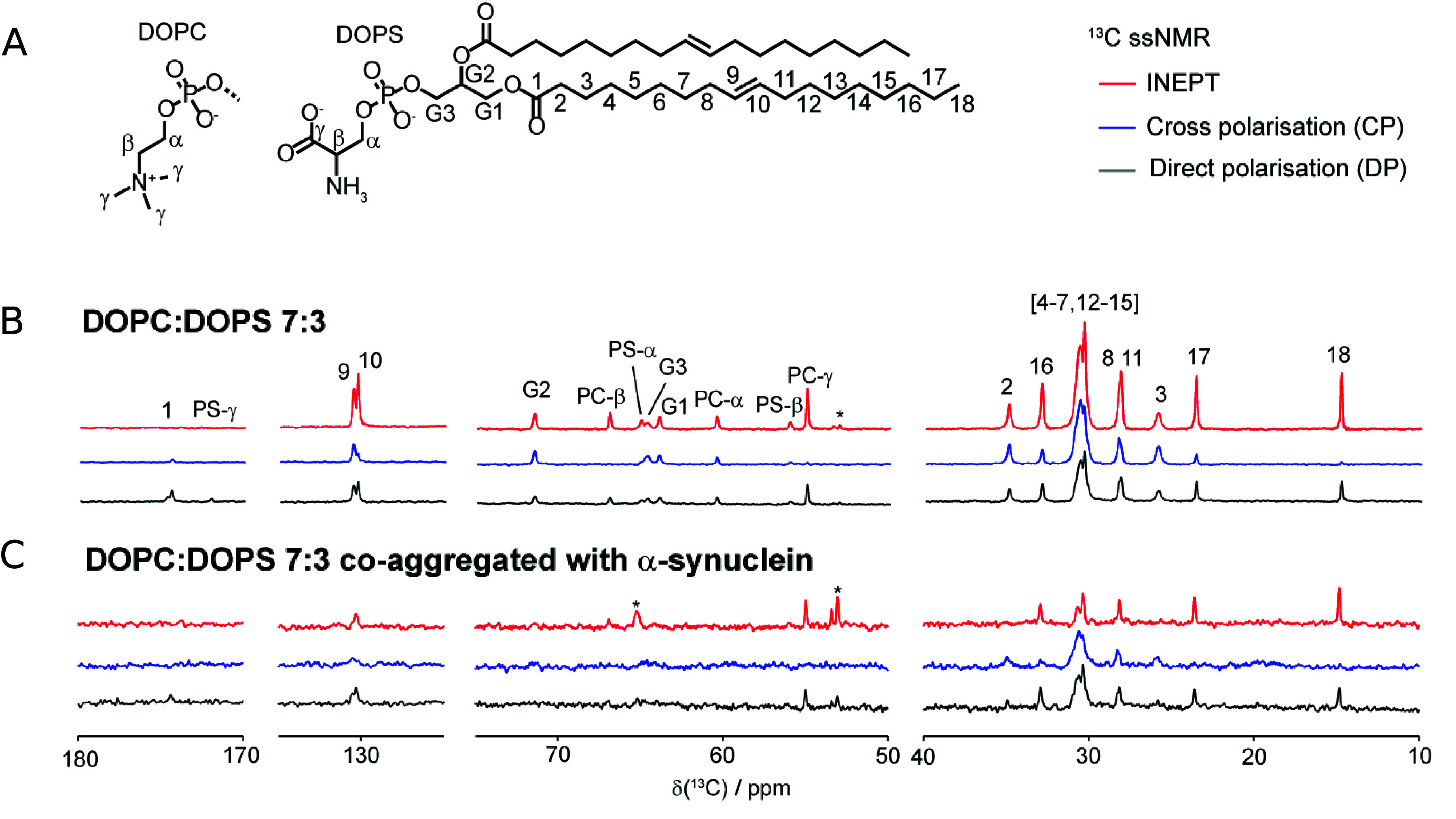
α-Synuclein coaggregation with lipids studied with solid state NMR. (A) Chemical structures of DOPC and DOPS lipids. Polarization transfer solid state NMR on lamellar phase lipids (B) and lipids coaggregated with α-synuclein (C). The comparison of the DP (Direct Polarization), CP (Cross-Polarization), and INEPT (insensitive Nuclei Enhanced by Polarization Transfer) spectra provides information on the molecular mobility of lipid molecules within protein–lipid coaggregates. In a CP and INEPT experiments, the signals from segments of low and high molecular mobility are enhanced, respectively. Reproduced from ref (30). Copyright 2013 American Chemical Society.

The above-described results suggest that the membrane not only plays the role of a surface in the aggregation process but also that lipid molecules may be incorporated into protein aggregates. Indeed, ^13^*C* Magic Angle Spinning (MAS) NMR spectra of the α-synuclein aggregates formed in the presence of lipid vesicles contain resonances corresponding to lipids (Figure 8), which implies that the lipid molecules are incorporated into the amyloid fibrils.^17,89−92^

Even though the fast kinetics of α-synuclein aggregation in the presence of membranes composed of only DLPS and DMPS, make it a convenient system for experimental studies, it should be emphasized that biological membranes typically contain lower amounts of charged lipids (up to 30 mol %) and lipid species with longer hydrocarbon chains (18–22 carbon atoms).^73,93^ It was recently shown that SUVs composed of DMPS undergo fragmentation upon α-synuclein adsorption,^94^ which does not occur for SUVs composed of lipid species with longer acyl chains such as DOPC and DOPS.^57^ Thus, in the case of membranes composed of lipids with short chains, there may be alternative routes to acceleration of α-synuclein aggregation than in the case of lipid species with longer chains.

## Conclusions and Open Questions

The end state of a system composed of α-synuclein and phospholipid membranes depends not only on the total protein concentrations and lipid membrane composition but also critically on the protein-to-lipid molar ratio. In the regime of membrane excess, the protein is not randomly distributed over the membrane but adsorbs in patches. This is a manifestation of the strong positive cooperativity of α-synuclein adsorption to phospholipid membranes, which means that a protein molecule prefers to associate at a location on the membrane where there is already α-synuclein adsorbed as opposed to a bare membrane. Two questions that emerge naturally are what are the molecular determinants and driving forces of the cooperativity, and what is the functional significance of this phenomenon.

A key to the functional significance of cooperativity is the fact that α-synuclein adsorption to lipid membranes causes membrane deformation, which may proceed to membrane fission. α-synuclein clustering on membranes *in vivo* may thus induce a curvature in the covered patch. This is expected to lead to membrane budding. Cooperative adsorption leads to a locally high surface concentration of the protein also in conditions with overall low protein concentration and provides an efficient way to induce membrane deformation on a specific place on the membrane. Factors that affect the strength of the membrane interaction of α-synuclein and its cooperativity may thereby modulate membrane deformation. Elucidating the molecular mechanisms responsible for the cooperativity is expected to provide important insights into how membrane deformation processes are modulated *in vivo*.

The discovery of the cooperativity of α-synuclein binding to lipid membranes and its connection with membrane deformation may shed new light on the healthy function of α-synuclein. Neurotransmitter release at the synapse involves trafficking of synaptic vesicles, their merging with the membrane of the synapse, release of their contents, and recycling of the vesicles. These are processes involving membrane remodeling, which for proper systemic function must occur when triggered by specific stimuli at a specific location. Cooperative protein association, which induces membrane deformation, is likely to play a key role in orchestration of these phenomena.

In this Perspective, we discussed the effect of monomeric α-synuclein adsorption to lipid membranes on the membrane morphology. *In vivo*, it is likely that the α-synuclein-membrane interaction is modulated by other proteins. In fact, α-synuclein was shown to interact with syntaxin-1, SNAP-25, and synaptobrevin-2,^14^ which are proteins involved in synaptic vesicle fusion and with calmodulin which is believed to regulate secretory processes at the synapse.^95^ α-Synuclein was also shown to promote clathrin-mediated endocytosis of synaptic vesicles.^96^ We refer readers interested in these aspects to the review by Eliezer and Snead.^97^ The molecular mechanisms of the role of α-synuclein in these contexts remain to be understood, while the insights from experimental studies of the α-synuclein effect on membranes in the absence of other proteins are expected to form the basis for understanding the complex interplay of many proteins *in vivo*.

The second theme of this Perspective, the effect of lipid membranes on α-synuclein amyloid formation, relates to the molecular mechanisms underlying Parkinson’s Disease pathology. Here one needs to consider the free energy landscape of all protein states (free/membrane-associated protein, protein fibril, protein–lipid coaggregate). Perturbations of this free energy landscape by changes in solution conditions, mutations, and PTMs as well as interactions with other proteins may facilitate or inhibit amyloid formation. The efficacy of putative inhibitors may also be dampened in the presence of membranes due to partitioning of the compounds into the bilayer.^98^ We also highlight the experimental evidence that the protein-covered membrane interface plays important roles in the molecular mechanism of membrane-templated amyloid formation.^26^ Here, we note that the interactions between membranes and α-synuclein oligomers may play important roles *in vivo*, as recently reviewed.^99^

Finally, the lipid–protein interactions leading to protein–lipid coaggregation may be crucial to the understanding of the formation of pathological inclusions in the brains of Parkinson’s Disease patients, which have been shown to contain both α-synuclein amyloid fibrils and lipids.^17,100^ Studies of membrane effects on aggregate structure may shed light on the effect of lipids on the stability of α-synuclein amyloid fibrils and contribute to a better understanding of the thermodynamics of the system.
